# Dietary component isorhamnetin is a PPARγ antagonist and ameliorates metabolic disorders induced by diet or leptin deficiency

**DOI:** 10.1038/srep19288

**Published:** 2016-01-18

**Authors:** Yu Zhang, Ming Gu, Wujie Cai, Lijing Yu, Li Feng, Lu Zhang, Qingqing Zang, Yahui Wang, Dongshan Wang, Hui Chen, Qingchun Tong, Guang Ji, Cheng Huang

**Affiliations:** 1 School of Pharmacy, Shanghai University of Traditional Chinese Medicine, 1200 Cailun Road, Shanghai 201203, China; 2 Brown Foundation Institute of Molecular Medicine and Programs in Neuroscience and Biochemistry, University of Texas Medical School at Houston, TX 77030, USA; 3 Institute of Digestive Disease, Longhua Hospital, Shanghai University of Traditional Chinese Medicine, Shanghai 201203, China

## Abstract

Studies on peroxisome proliferator-activated receptor (PPAR)-γ ligands have been focused on agonists. However, PPARγ activation may induce obesity and nonalcoholic fatty liver disease (NAFLD), one of the most challenging medical conditions. Here, we identified that isorhamnetin, a naturally occurring compound in fruits and vegetables and the metabolite of quercetin, is a novel antagonist of PPARγ. Isorhamnetin treatment inhibited the adipocyte differentiation induced by the PPARγ agonist rosiglitazone, reduced obesity development and ameliorated hepatic steatosis induced by both high-fat diet treatment and leptin deficiency. Our results suggest that dietary supplement of isorhamnetin may be beneficial to prevent obesity and steatosis and PPARγ antagonists may be useful to treat hepatic steatosis.

Metabolic syndrome, as a result of unbalanced energy homeostasis and obesity, is a complex disorder including obesity, hypertension, insulin resistance, hyperlipidemia. Metabolic disorders in turn increase the risk of atherosclerosis and diabetes. Currently, there is no satisfying therapeutic strategy for metabolic syndrome.

PPARγ is a ligand-activated transcription factor and is thought to be a key regulator of lipogenesis, fatty acid storage and glucose metabolism^1^,^2^. PPARγ plays a crucial role in the development of hepatic steatosis. One feature of steatotic liver is increased PPARγ expression, which activates lipogenic genes^3^,^4^. The PPARγ agonist rosiglitazone, a potent insulin sensitizer, produces a marked worsening in oxidative stress and liver steatosis, indicating that the activation of PPARγ may induce steatosis^5^. On the other hand, the PPARγ heterozygous mouse showed resistance to diet-induced obesity, insulin resistance and fatty liver^6^,^7^. Also, the Pro12Ala polymorphism in human PPARγ2 lowers the activity of PPARγ and reduces body mass index and blood glucose^8^,^9^. PPARγ antagonists have been shown to have anti-obesity and anti-diabetic activity^10^,^11^,^12^,^13^, suggesting that the inhibition of PPARγ activity may be beneficial to prevent obesity, steatosis and other metabolic disorders.

Epidemiological studies indicate that consumption of foods rich in flavonoids may reduce the incidence of lifestyle diseases such as coronary heart disease^14^,^15^. Isorhamnetin (Fig. 1a), also called 3-O-methylquercetin, is a naturally occurring flavonoid in many vegetables and fruits^16^,^17^,^18^,^19^, and is the metabolite of quercetin^20^,^21^. It is reported that isorhamnetin has antioxidant, anti-inflammatory, and chemopreventive activities, and protects ventricular myocytes from ischemia and reperfusion injury^22^,^23^,^24^,^25^,^26^. Recently, isorhamnetin has also been showed to be a PPARγ agonist^27^. However, other reports showed that isorhamnetin inhibits the differentiation and the adipogenesis of 3T3-L1 adipocytes^28^,^29^, and isorhamnetin glycosides lower body weight, fasting blood glucose and lipid profile in diet-induced obese (DIO) C57BL/6 mice^30^, which are opposed to the function of isorhamnetin as a PPARγ agonist.

Here, we provided the evidence that isorhamnetin antagonized the effect induced by rosiglitazone and prevented metabolic dysfunction in both diet-induced and leptin-deficient obese mice, thereby identifying isorhamnetin as a novel natural PPARγ antagonist and a potential treatment option for metabolic disorders associated with obesity.

## Results

### Isorhamnetin inhibits 3T3-L1 adipocyte differentiation

**Figure 1:**
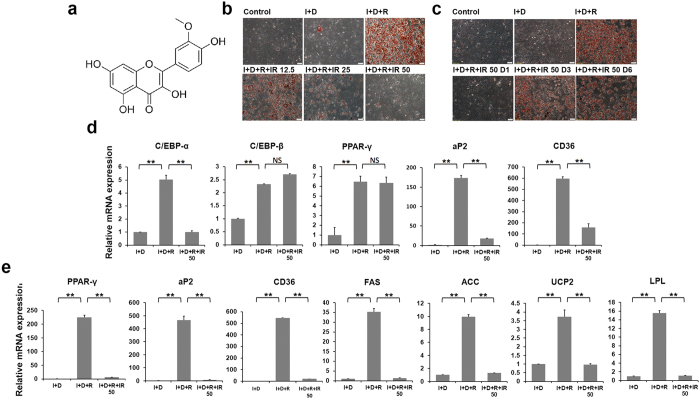
Isorhamnetin suppresses 3T3-L1 adipocyte differentiation and adipogenesis related gene expression. (**a**) Structure of isorhamnetin. (**b**) Isorhamnetin suppresses 3T3-L1 adipocyte differentiation induced by differentiation medium. (**c**) The inhibition of adipocyte differentiation is time-dependent. I + D + R + IR 50 D1-D6: 50 μM of IR was added to medium on day 1, day 3 and day 6. (**d,e**) Gene expression profile at day 0 (**d**) and day 6 (**e**) in differentiated 3T3-L1 cells. Control: growth medium. I + D: insulin and dexamethasone. I + D + R: insulin, dexamethasone and rosiglitazone. IR: isorhamnetin. Data are presented as means ± SEM (n = 3). *P < 0.05, **P < 0.01.

To identify PPARγ ligands, we screened about 1000 natural compounds using 3T3-L1 adipocyte differentiation model induced by insulin, dexamethasone and PPARγ agonist rosiglitazone. We found that isorhamnetin markedly inhibited rosiglitazone dependent 3T3-L1 adipocyte differentiation (Fig. 1b). Isorhamnetin is the metabolite of quercetin and an o-methylated flavonol that is abundant in sea-buckthorn, a fruit used as food and a nutritional supply. Quercetin exists in many fruits and vegetables, and has been reported to have therapeutic roles in various diseases, including metabolic disorders^21^,^31^,^32^. As seen in Fig. 1b, isorhamnetin treatment remarkably inhibited adipocyte differentiation at a concentration of 12.5 μM, and thoroughly blocked it at 50 μM. The inhibitory effect of differentiation was less potent when isorhamnetin was added at day 3 of differentiation or later (Fig. 1c). The results indicate that isorhamnetin inhibits early stage of adipocyte differentiation.

To examine the transcription changes associated with adipocyte differentiation, we measured expression of key genes known to be important for adipocyte differentiation at both early and late stages. At early stage, we observed reduced expression of the adipogenesis markers adipose fatty acid-binding protein 2 (aP2), cluster of Differentiation 36 (CD36), and transcription factor CCAAT-enhancer-binding proteins (C/EBP) α, but not of transcription factors C/EBPβ and PPARγ, known to control early stage differentiation, suggesting that isorhamnetin may inhibit the transactivation of PPARγ (Fig. 1d). At late stage, we observed a more complete inhibition of the expression of PPARγ and its target genes aP2, CD36, uncoupling protein 2 (UCP-2), lipoprotein lipase (LPL), acetyl coenzyme A carboxylase (ACC) and fatty acid synthase (FAS) (Fig. 1e).

### Isorhamnetin antagonizes PPARγ transactivity

**Figure 2:**
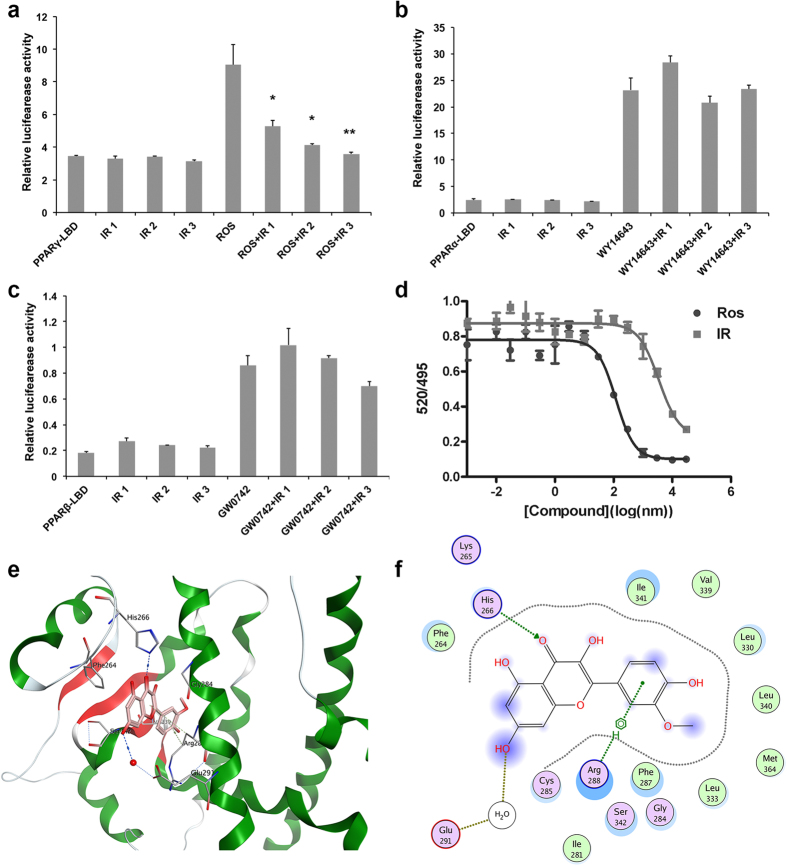
Isorhamnetin is a natural antagonist of PPARγ. (**a**) Isorhamnetin specially inhibits the transcriptional activity of PPARγ, but not PPARα (**b**) and PPARβ (**c**). IR1, IR2, IR3: 10, 25 and 50 μM of IR. (**d**) Binding of isorhamnetin and rosiglitazone on PPARγ-LBD in a competitive TR-FRET assay. (**e**) The structure of the complex of the PPARγ LBD and isorhamnetin by molecular docking. (**f**) The 2D-interaction map of the complex of the PPARγ LBD and isorhamnetin. IR: isorhamnetin. Data are presented as means ± SEM (n = 3). *P < 0.05, **P < 0.01.

PPARγ plays a critical role in adipocyte differentiation and fat deposition, which is expressed predominantly in adipose tissue and liver tissue. Therefore, we speculated that isorhamnetin suppresses adipocyte differentiation by directly inhibiting the PPARγ pathway. To test this hypothesis, we performed a reporter assay using a Gal4-PPARγ ligand-binding domain (LBD) expression plasmid and a UAS reporter plasmid. Figure 2a shows that isorhamnetin inhibited transcriptional activity of PPARγ induced by rosiglitazone, but not that of PPARα,β/δ (Fig. 2b,c) or liver X receptor (LXR) α/β, in a dose-dependent manner, indicating that isorhamnetin is a specific antagonistic ligand of PPARγ. We further used Time-Resolved Fluorescence Resonance Energy Transfer (TR-FRET) to confirm the binding of the compound to PPARγ. Isorhamnetin displaced the labelled PPARγ-ligands in a dose-dependent manner with an IC50 value of 3.5 μM (K_i_ = 1.27 μM), which was higher than the IC_50_ value of 0.12 μM (K_i_ = 0.043 μM) of the potent ligand of PPARγ, rosiglitazone (Fig. 2d).

To obtain further insights into the interaction of isorhamnetin with PPARγ, we analyzed the nature of binding between the PPARγ LBD and isorhamnetin using molecular docking (Fig. 2e). The PPARγ ligand binding site contains a large cavity and enclosed helices and beta-strands. The cavity is Y-shaped and includes an entrance and two pockets. Most known agonists (e.g. thiazolidinediones, TZDs) compete with endogenous ligands for a canonical ligand-binding pocked (CLBP) of LBD, which consist of four conserved polar residues in arm I and hydrophobic part of the entrance in arm II. Based on the affinity scoring and binding pose, isorhamnetin occupies the same pocket as rosiglitazone. But unlike rosiglitazone and the antagonist GW9662, the binding between isorhamnetin and PPARγ mainly takes place in the hydrophobic part of the entrance. The 2D-interaction map of best ranking pose was shown in Fig. 2f. The nucleus of isorhamnetin is composed of chromone and phenyl, which are both hydrophobic. The hydrophobic nucleus of isorhamnetin helps anchor into the hydrophobic pocket. There is an H-bond between the carbonyl group at C-4 position and His266. This H-bond could enhance the affinity of isorhamnetin because of the high bond strength. In addition, the center of phenyl with a negative charge would have weak electric interactions with the positively charged Arg288. Combined with reporter-gene results, isorhamnetin may replace the H-bond binding of agonist and push the agonist out of bound position, to block activation of AF-2. Taken together, isorhamnetin is a novel naturally occurring PPARγ antagonist.

### Isorhamnetin ameliorates metabolic disorders induced by diet

**Figure 3:**
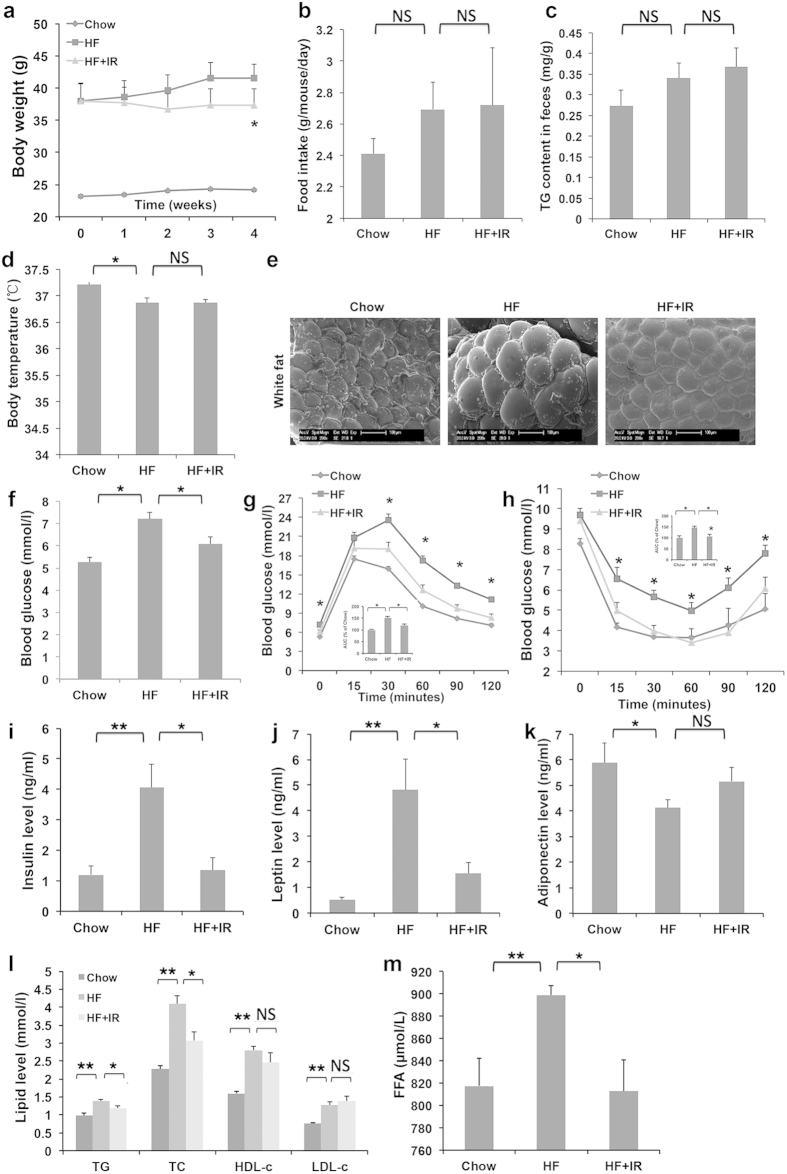
Isorhamnetin reduces body weight, insulin resistance and serum lipid level in DIO mice. (**a**) Body weight. (**b**) Food intake amount. (**c**) Fecal triglyceride level. (**d**) Body temperature. (**e**) The mass of white adipose tissue. (**f**) Fasting blood glucose level. (**g**) Glucose tolerance test (GTT). The area under the curve (AUC) was calculated from the GTT graph. (**h**) Insulin tolerance test (ITT). The area under the curve (AUC) was calculated from the ITT graph. (**i**) Serum insulin level. (**j**) Serum leptin level. (**k**) Serum adiponectin level. (**l**) Serum TC, TC, LDL-c and HDL-c. (**m**) Plasma free fatty acid (FFA) levels. The mice were treated for 4 weeks. IR: isorhamnetin. Data are presented as means ± SEM (n = 8). *P < 0.05, **P < 0.01. NS: No significance.

As isorhamnetin inhibited PPARγ-dependent adipocyte differentiation, we next investigated the effect of isorhamnetin on obesity and metabolic disorders induced by high-fat diet. Three month high-fat diet feeding increased the body weight in C57/BL6 mice^13^ (Fig. 3a). Four-week isorhamnetin treatment remarkably reduced body weight of mice (Fig. 3a), which was not associated with changes in food intake, fecal TG or body temperature (Fig. 3b–d). We examined the structure of white adipocytes using scanning electron microscopy. Consistently, isorhamnetin reduced the cell size of abdominal visceral white adipocyte tissue (WAT), compared with that of high fat (HF) control mice (Fig. 3e), demonstrating that isorhamnetin significantly blocked the HF-induced increase in fat mass.

Insulin resistance is accompanied by the development of obesity. Therefore, we investigated whether isorhamnetin could improve insulin resistance in DIO mice. After isorhamnetin treatment, the fasting glucose levels were lower and glucose tolerance were improved at 30, 60, 90 and 120 min following intraperitoneal administration of glucose (a 32% decrease in area under curse (AUC)) (Fig. 3f,g). Similarly, blood glucose levels were lower at 15, 30, 60, 90 and 120 min after intraperitoneal injection of insulin (a 39% decrease in AUC) (Fig. 3h). Furthermore, serum insulin levels in DIO mice were elevated, which was attenuated by isorhamnetin treatment (Fig. 3i), indicating that isorhamnetin improved insulin resistance.

Leptin and adiponectin are adipocytokines that affect glucose and lipid metabolism^33^,^34^. Leptin may contribute significantly to the fatty liver improvement effects in *PPAR*γ ^+/−^ mouse^35^. We thus tested whether isorhamnetin treatment could regulate leptin and adiponectin levels. Isorhamnetin significantly reduced plasma concentrations of leptin by high-fat diet feeding (Fig. 3j). In contrast, isorhamnetin did not significantly change adiponectin levels, but rather showed a tendency to increase (Fig. 3k).

Since dyslipidemia and hypercholesterol are often induced by obesity, we assayed the serum lipid profiles. Isorhamnetin treatment significantly decreased the serum TG, total cholesterol (TC) and free fatty acid (FFA) levels. However, the serum low-density lipoprotein cholesterol (LDL-c) and high- density lipoprotein cholesterol (HDL-c) levels were not markedly affected by isorhamnetin treatment (Fig. 3l,m). These results indicate that isorhamnetin may also prevent HF diet-induced dyslipidemia.

### Isorhamnetin ameliorates metabolic dysfunction by leptin deficiency

**Figure 4:**
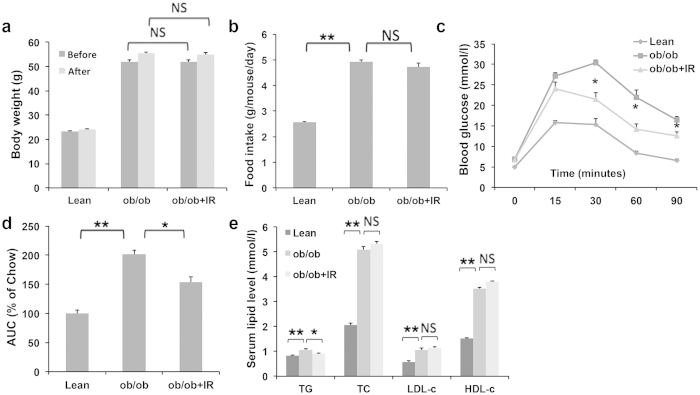
Isorhamnetin treatment ameliorates metabolic profiles in *ob/ob* mice. (**a**) Body weight of *ob/ob* mice. (**b**) Food intake amount. (**c**) Glucose tolerance test (GTT). (**d**) The AUC of each group from the GTT graph. (**e**) Serum TC, TG, LDL-c and HDL-c content. The mice were treated for 2 weeks. IR: isorhamnetin. Data are presented as means ± SEM (n = 8). *P < 0.05, **P < 0.01. NS: No significance.

We then tested whether isorhamnetin could ameliorate metabolic disorders in leptin deficient *ob*/*ob* mice. As shown in Fig. 4a,b, daily treatment with isorhamnetin did not induce any changes in body weight, but did improve glucose tolerance at 30, 60 and 90 min and glucose AUC compared with the untreated group (Fig. 4c,d). Isorhamnetin treatment also markedly reduced the serum TG contents. The serum TC, LDL-c, and HDL-c were not affected by isorhamnetin treatment (Fig. 4e).

### Isorhamnetin ameliorates hepatic steatosis

**Figure 5:**
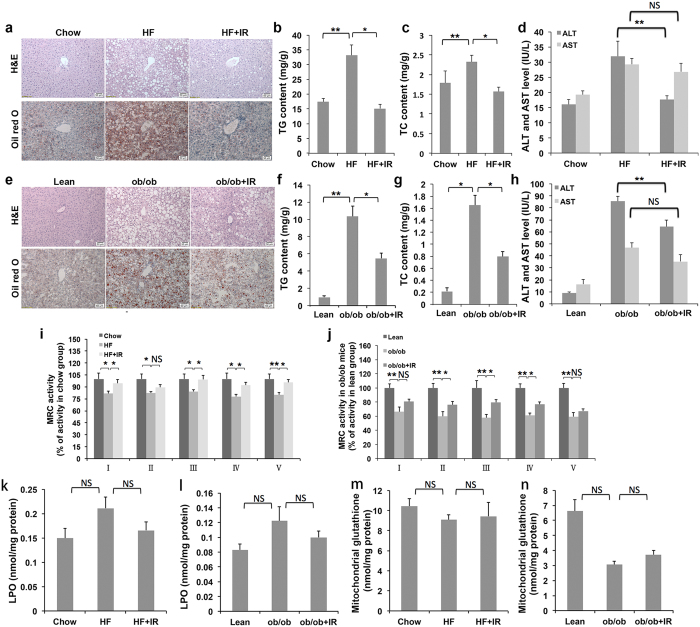
Isorhamnetin ameliorates steatosis in DIO and *ob/ob* mice. (**a**) Histological analysis of liver after the 4 week treatment in obese mice (×200). Scale bars represent 50 μm. (**b,c**) TG and TC levels in the liver of obese mice. (**d**) ALT and AST levels in serum of obese mice. (**e**) Histological analysis of the liver after 2 week treatment in *ob/ob* mice (×200). (**f,g**) TG and TC levels in the liver of *ob/ob* mice. (**h**) ALT and AST levels in serum of *ob/ob* mice. (**i**) Mitochondrial respiratory chain activity in obese mice. (**j**) Mitochondrial respiratory chain activity in *ob/ob* mice. (**k**) Liver LPO (lipid peroxide) content in obese mice. (**l**) Liver LPO content in *ob/ob* mice. (**m**) Liver glutathione content in obese mice. (**n**) Liver glutathione content in *ob/ob* mice. IR: isorhamnetin. Data are presented as means ± SEM (n = 8). *P < 0.05, **P < 0.01. NS: No significance.

Obese mice usually develop hepatic steatosis due to excess storage of fat in liver^36^. To investigate the effect of isorhamnetin on hepatic steatosis, we examined fat content and lipid profile in the liver of isorhamnetin-treatment DIO mice. Histological examination indicated that the chow mice had normal liver histology, whereas the DIO mice exhibited a marked accumulation of lipid droplets, and showed balloon degeneration of hepatocytes and microvesicular and macrovesicular steatosis^13^ (Fig. 5a). Out of total 8 control group mice examined, one mouse displayed grade 2, and seven displayed grade 3 steatosis that showed macrovesicular droplets in 50% to 70% of hepatocytes. Daily treatment with isorhamnetin notably reversed the tissue structure and improved hepatic steatosis in the mice (Fig. 5a). Among 8 mice treated with isorhamnetin, only 2 mice exhibited grade 2 steatosis and the left had grade 1 steatosis. In contrast to the obese group, isorhamnetin reduced the accumulation of liver triglycerides and cholesterol by approximately 50% (Fig. 5b,c). Aminotransferase levels, a marker of liver damage^37^, were remarkably increased in DIO mice compared to normal control mice. As shown in Fig. 5d, serum levels of alanine aminotransferase (ALT) were significantly decreased in DIO mice by isorhamnetin treatment. However, serum aspartate aminotransferase (AST) levels remained unchanged.

Similarly, Oil red O and HE staining showed that the liver sections of *ob/ob* mice displayed a severe accumulation of fat droplets, 10% microvesicular steatosis and 90% macrovesicular steatosis and all mice exhibited grade 3 steatosis compared to that seen in chow mice^13^ (and Fig. 5e). Isorhamnetin treatment markedly reduced the steatosis in the liver (Fig. 5e) and macrovesicular steatosis was reduced 50%, though only 3 mice was exhibited grade 2. TG and TC contents in the livers of isorhamnetin treated mice also reduced by approximately 50% (Fig. 5f,g). Isorhamnetin remarkably reduced the levels of serum ALT, but not AST in *ob*/*ob* mice (Fig. 5h). Collectively, the data suggest that isorhamnetin may be beneficial to prevent hepatic steatosis in the obese mice.

Mitochondrial dysfunction, particularly which in mitochondrial respiratory chain (MRC) activity, is a critical phase in the pathogenesis of NAFLD^38^. PPARγ agonists have been shown to decrease the MRC complex I activity and increase oxidative stress, thereby exacerbating liver steatosis^5^,^39^. We therefore studied the effects of isorhamnetin on the activity of MRC complexes. The activity of complexes (I-V) in liver tissue was significantly reduced in both DIO and *ob/ob* mice, compared to the control. Administration with isorhamnetin resulted in a remarkable increase in the activity of the MRC complexes I, III, IV and V in DIO mice, and I, II, III and IV in *ob*/*ob* mice, indicating that isorhamnetin may ameliorate hepatic steatosis via the activation of mitochondrial MRC activities in obese mice (Fig. 5i,j).

Since NAFLD includes hepatic steatosis, Non-alcoholic steatohepatitis, fibrosis and cirrhosis^4^, we investigated lipid peroxide and mitochondrial glutathione levels in mouse liver. As shown in Fig. 5k–n, isorhamnetin treatment did not change lipid peroxide and mitochondrial glutathione contents in the liver of the obese mice.

### Isorhamnetin suppresses the expression of PPARγ downstream genes

**Figure 6:**
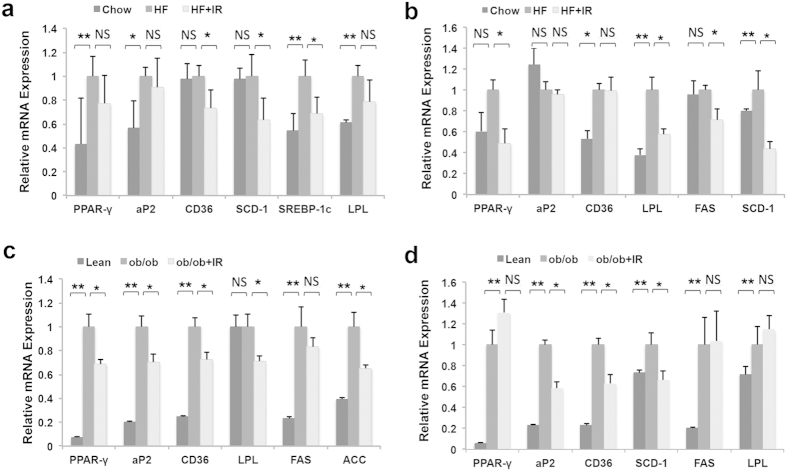
IR inhibits the mRNA expression of the PPARγ target genes. (**a,b**) RT-PCR analysis in white adipose tissue (**a**) and liver tissue (**b**) of obese mice. (**c,d**) RT-PCR analysis in white adipose tissue (**c**) and liver tissue (**d**) of *ob/ob* mice. IR: isorhamnetin. β-actin was used as for normalizing the mRNA levels. Data are presented as means ± SEM (n = 8). *P < 0.05, **P < 0.01 *versus* HF group or *ob/ob* group, NS: No significance.

To confirm the mechanism of isorhamnetin action, we analyzed the expression of PPARγ regulating genes in the liver and WAT of the mice. The results showed that HFD feeding increased the mRNA levels of PPARγ, aP2, sterol regulatory element-binding protein (SREBP) 1c and LPL in WAT (Fig. 6a), and CD36, LPL and stearoyl-CoA desaturase (SCD) 1 (Fig. 6b) in liver tissue in DIO mice compared with chow diet feeding. After IR treatment, the expression of CD36, SCD-1 and SREBP -1c in WAT (Fig. 6a) and PPARγ, LPL, FAS and SCD-1 in liver tissue was remarkably decreased in DIO mice (Fig. 6b).

In *ob*/*ob* mice, the mRNA expression of PPARγ, aP2, CD36, LPL, FAS and Acetyl-CoA carboxylase (ACC) in WAT (Fig. 6c) and PPARγ, aP2, CD36, SCD-1, FAS and LPL in liver (Fig. 6d) was remarkably up-regulated compared with lean mice. Similarly, IR treatment notably reduced the expression of PPARγ, aP2, CD36, LPL and ACC in WAT (Fig. 6c) and aP2, CD36 and SCD1 in liver (Fig. 6d) of the *ob*/*ob* mice. Collectively, these results suggest that isorhamnetin may reduce fat through the inhibition of PPARγ transcription activity.

## Discussion

Isorhamnetin has been reported to be a PPARγ agonist^27^. In the present study, we demonstrated that isorhamnetin is a naturally occurring PPARγ ligand. Reporter assay using PPARγ-LBD-GAL4 system indicated that isorhamnetin inhibited the rosiglitazone induced PPARγ transactivity, but had no effect on PPARα, PPARβ/δ and LXRα and β transactivities. This may be mediated by competition of isorhamnetin and rosiglitazone with same binding site at ligand binding domain of PPARγ. The FRET binding assay supported that isorhamnetin is a ligand of PPARγ because it competes with labelled PPARγ-ligands to bind to the human PPARγ-LDB in a high affinity with IC_50_ of 3.5 μM (Fig. 2d). Also, molecular docking assay suggested that isorhamnetin may interact with PPARγ LBD, where hydrogen bonds at Cys285, His449 and Met364 are predicted to be formed, in addition to extensive hydrophobic interactions (Fig. 2e,f). The predicted binding mode revealed that the interactions between isorhamnetin and the PPARγ LBD is similar to the interaction between PPARγ and its known agonists, which suggests that isorhamnetin is a ligand of PPARγ.

However, our data do not support that isorhamnetin is an agonist of PPARγ. Isorhamnetin suppresses PPARγ agonist rosiglitazone induced adipocyte differentiation of 3T3-L1 cells. Many previous reports have indicated that rosiglitazone and other PPARγ agonist could induce the differentiation of 3T3-L1 adipocyte^40^,^41^,^42^, while antagonist of PPARγ may inhibit 3T3-L1 differentiation^10^,^13^. In the present study, we found that isorhamnetin inhibited the adipocyte differentiation with a dose dependent manner. Meanwhile, isorhamnetin also attenuated PPARγ mRNA level and its target genes, which may be increased by PPARγ agonist. These effects were further confirmed in the liver and WAT of both DIO and *ob/ob* mice. The results are in agreement with the previous reports that isorhamnetin inhibits the 3T3-L1 adipocyte differentiation and suppress the PPARγ target gene expression^28^,^29^,^43^. Thus, most likely, isorhamnetin is a PPARγ antagonist.

PPARγ antagonists have been reported to ameliorate the metabolic disorders in the animals^10^,^12^,^13^. A previous study showed that heterozygous PPARγ-deficient mice were resistant to the development of insulin resistance induced by a HF diet^6^. Thus, the suppression of PPARγ transactivity may protect metabolic disorders such as obesity, fatty liver and insulin resistance. In the present study, isorhamnetin significantly ameliorated the insulin resistance, fatty liver, hyperlipidemia and other metabolic disorders as other PPARγ antagonist did. Interestingly isorhamnetin attenuated the serum insulin and leptin levels in DIO mice, indicating an improvement of insulin resistance and leptin resistance. Surprisingly, isorhamnetin did not reduce body weight in leptin deficiency *ob*/*ob* mice, indicating that the weight-reducing effect of isorhamnetin may be dependent on the intact leptin signaling as that in heterozygous PPARγ-deficient mice^6^.

Quercetin has been reported to reduce hepatic steatosis^21^,^44^ and obesity related non-alcoholic steatohepatitis^45^,^46^ in DIO mice. Administration of quercetin to mice resulted in high levels of isorhamnetin, a metabolite of quercetin, in the liver^44^. However, it is not clear whether isorhamnetin plays a role in the treatment of fatty liver. NAFLD is highly associated with obesity and insulin resistance. In the present study, we observed that isorhamnetin attenuated fat droplets and improved liver morphology in DIO mice in a similar fashion to other PPARγ antagonists, suggesting that isorhamnetin can be used to prevent hepatic steatosis. Low MRC contents are linked to the pathogenesis of hepatic steatosis. MRC complexes could be reduced by rosiglitazone or pioglitazone induced PPARγ activation, which may be responsible for the exacerbation of hepatic steatosis^37^. Isorhamnetin increased the levels of MRC complexes I, III, IV and V in DIO mice, and complex II, III, IV in *ob*/*ob* mice. Combined with the gene expression data in liver, these data indicate that isorhamnetin may alleviate hepatic steatosis via supressing PPARγ transactivity and reducing the MRC contents in the liver of obese mouse.

In conclusion, we identified that isorhamnetin is a dietary source of PPARγ antagonist. Isorhamnetin reduced body weight, ameliorated insulin resistance and alleviated hepatic steatosis in obese mice via the suppression of PPARγ transactivity. Our data suggest that dietary element isorhamnetin may be beneficial to obesity and steatosis prevention.

## Material and Methods

### Compounds

Isorhamnetin of 98% purity was purchased from Zelang Pharmaceutical Co., Ltd. (Nanjing, China). Rosiglitazone was from Enzo Life Science (Lausen, Switzerland). WY14643 and GW0746 were obtained from Sigma (St. Louis, MO, USA). All compounds were dissolved in dimethylsulfoxide (DMSO) for the *in vitro* studies.

### Differentiation

3T3-L1 pre-adipocyte were cultured in Dulbecco’s modified eagle medium (DMEM) high glucose containing 1% Penicillin-Streptomycin (PS) and 10% fetal bovine serum (FBS, Hyclone, Logan, UT, USA). To induce differentiation, 2-days post confluence preadipocytes (day 0), the culture medium was exchanged with DMEM supplemented with 1% PS and 10% FBS and 1 μM dexamethasone (DEX, Sigma-Aldrich)/10 μg/ml insulin (Sigma-Aldrich)/10 μM rosiglitazone (ROS). After 48 h, the cells were then maintained for another 2 days in culture media and insulin. Thereafter, the cells were maintained in culture medium and the medium was changed every 2 days. At the beginning of differentiation (day 0), isorhamnetin was added to the culture medium differentiation with the 12.5 μM, 25 μM and 50 μM. To examine the effect of Isorhamnetin on adipocyte differentiation at different stage, the adipocytes were treated with Isorhamnetin at a concentration of 50 μM. At RT-PCR experiment, Cells were treated with isorhamnetin at day 0 and day 6.

### Oil red staining

For determining the adipogenesis, the cells were stained with Oil Red O at day 6. Briefly, cells were washed gently with phosphate-buffer saline (PBS) and the fixed with 10% formalin for 10 min. The fixed cells were stained with filtered Oil-red O solution for 30 min at 50 °C. Photographs were taken with an Olympus (Tokyo, Japan) microscope.

### Transient transfection and luciferase reporter assays

HEK 293T cells were seeded in 48-well plates 24 h prior to transfection and were cultured in DMEM supplemented with 1% PS and 10% FBS. Transfections were performed with pCMX-Gal-mPPARα,β,γ LBD, pCMX-Gal, and the Gal4 reporter vector MH100-4-TK-Luc using FuGENE-HD (Roche, Mannheim, Germany) according to the manufacturer’s protocol. After 24 h, cells were treated with compounds (isorhamnetin: 12.5, 25 and 50 μM, rosiglitazone, WY14643 and GW0742: 10 μM) for 24 h. Luciferase activity was then determined using the Dual-Luciferase Reporter Assay System (Promega, USA) after lysing the cells. For normalization, renilla plasmids were co-transfected into 293T cells along with the plasmids containing promoter-luciferase reporter gene. All transfections were performed in triplicate.

### Total RNA isolation, cDNA preparation, and quantitative real-time PCR

Real-time PCR was carried out as previous described^13^. Briefly, total RNA from 3T3-L1 adipocytes and liver and adipose tissue was isolated using the Trizol reagent (TaKaRa, Japan). Then, 3 μg sample of total RNA was subjected to first-strand cDNA synthesis using the cDNA synthesis kit (Thermo, America) according to the manufacturer’s instructions. Real-time quantitative PCR (SYBR green) analysis was performed using the ABI step one plus Real-time PCR system (Applied Biosystems, Foster City, CA) with primer sequences as listed in Table 1. All gene expression was normalized using beta-actin as a reference gene. Target gene levels were presented as a ratio of levels intreated vs. corresponding control group. All experiments were performed in duplicate and all results were obtained from at least three independent experiments.

### Animals

All animals experiment fulfilled the requirement for the guidelines of the Institutional Animal Care and Use Committee of the Shanghai Institutes for Biological Sciences. The animal protocols used in this study were approved by the Shanghai University of Traditional Chinese Medicine (Approved Number: 2014007). Six-week-old female C57/BL6 mice were purchased from the SLAC Laboratory (Shanghai, China) and Eleven-week-old female C57BL/KsJ-*ob/ob* mice were obtained from the Model Animal Research Center of Nanjing University, and maintained at constant temperature and humidity with a 12:12-h light-dark cycle and permitted consumption of water ad libitum.

### Experimental Design and Diet

Eight mice were fed a low-fat diet (10% of calories derived from fat, Research Diets; D12450B) as chow group and other mice were fed a high-fat diet (60% of calories derived from fat, D12492, Research Diets, New Brunswick, NJ, USA) for 3 months to induce obesity. The obese mice were randomly divided into high-fat diet control (HF) and isorhamnetion treated group according to body weight as previous described^13^ (isorhamnetin was powered and mixed into HF diet at 1% (w/w) evenly). The mice were treated for 4 weeks. The *ob*/*ob* mice were randomly divided into two groups based on body weight and dosed each day with isorhamnetin (100 mg.kg^−1^ day^−1^) or saline using oral gavage (n = 8). Food intake and body weight were recorded every 2 days. Intraperitoneal glucose tolerance and insulin tolerance tests were measured after 4 weeks (DIO mice) and 2 weeks (*ob*/*ob* mice). At the end of the study, all mice were fasted overnight for 12 h and sacrificed, and then blood and tissues were collected for future measurements.

### Rectal Temperature Measurements

After treatment, the rectal temperatures of mice were assessed using a rectal probe connected to a digital thermometer (Physitemp, NJ, USA).

### Intraperitoneal Glucose Tolerance and Insulin Tolerance Test

C57/BL6 mice and *ob/ob* mice were fasted for 12 h, and injected with 1 g/kg glucose into the peritoneal cavity. Blood samples were obtained from the tail vein of each mouse and determined before and 15, 30, 60, 90 and 120 min. For insulin tolerance tests, mice were injected i.p. with insulin (0.75 U/kg body weight) (Sigma, St. Louis, MO). Glucose levels were examined before and 15, 30, 60, 90 and 120 min after injection.

### Serum Biochemical analysis

The levels of triglyceride (TG), total cholesterol (TC), HDL cholesterol (HDL-c), and LDL cholesterol (LDL-c) in the serum were measured using a Hitachi 7020 Automatic Analyzer (Hitachi, Ltd., Tokyo, Japan). The serum level of alanine transaminase (ALT) and aspartate aminotransferase (AST) were measured using a commercial assay kit (Nanjing Jiancheng Bioengineering Institute, China) according to the manufacturer’s protocol. Serum insulin (Salem, NH, USA), leptin (Oxon, UK) and adiponectin (Seoul, Korea) levels were examined using different commercial assay kits respectively based on an enzyme-linked immunosorbent method according the manufacturer’s instructions.

### Liver and fecal lipid content analysis

The liver tissues (about 50 mg) from each group were added to 10 volumes of pre-cold tissue lysis buffer (20 mM Tris-HCl pH 7.5, 150 mM NaCl, 1% Triton) and homogenized by sonication. The liver lysates were added to an equal volume of chloroform. The bottom of the tube is lipid layers. After dried and added 50 μl isopropyl alcohol to the tube, 2 μl of mixture was taken to measure TG and TC levels. Fecal lipids were extracted and measured as described above. The results were normalized by sample weight.

### Histological examinations of liver

The fresh liver samples were fixed in 4% paraformaldehyde, and divided into two sections. One section was frozen and embedded in O.C.T. compound (Sakura Finetek, Torrance, CA), sliced, and then stained with Oil red O. The other was embedded in paraffin and sectioned at 5 μm before being stained with hematoxylin and eosin. An Olympus microscope (Olympus, Tokyo, Japan) was used to visualize the H&E- and Oil Red O-stained tissue sections at ×200 magnification.

### Assay of metabolic enzyme and antioxidant indices in liver

Frozen liver tissues were homogenized in ice-cold saline buffer (1:9, wt/v) and homogenates were centrifuged at 2500 rpm for 10 minutes at 4 °C. The supernatants were collected and used for the assays of mitochondrial respiratory chain (MRC) activity, lipid peroxides and glutathione content. The assays for MRC activity (Bioyetime Biotechnology, China), lipid peroxide (Nanjing Jiancheng Bioengineering Institute, China) and glutathione (Beyotime Biotechnology, China) were determined with different kits, receptively according to the manufacturer’s protocol. The lipid peroxide and glutathione content were normalized by protein concentration.

### Scanning Electron Microscopy

The adipose tissue was fixed in 10% neutral formalin and then embedded in 1% osmium tetraoxide. A Philip XL-30 scanning electron microscope was used to observe the structure of fat tissue according to the above described methods.

### Nuclear receptor binding assays

To further identify PPARγ ligands, nuclear receptor binding assays were performed by a time-resolved fluorescence resonance energy transfer (TR-FRET) according to the manufacturer’s protocol (Lanthascreen, Invitrogen, Darmstadt, Germany). Briefly, when labeled PPARγ-ligands bind a labeled PPARγ ligand-binding domain, a high TR-FRET ratio (520/495) is observed. This energy transfer is detected, representing low TR-FRET ratio, once potential ligands displace the labeled PPARγ-ligands. Increasing the concentration of potential ligands leads to a more complete displacement of the labelled PPARγ-ligands and hence a lower TR-FRET signal. IC50 was calculated using GraphPad Prism 5.0 and Ki was fitted according to the equation: Ki = IC50/ (1 + [tracer]/K_D_). All experiments were performed in triplicate.

### Docking of isorhamnetin into PPARγ LBD

The co-crystalized structure (PDB Code: 2I4J) was prepared using MOE2012.10. The binding site for human PPARγ was characterized based on structural information derived from a variety of co-crystals (3B0R, 3T03, 2I4J, 2F4B, 2Q5S). The docking was carried out as previously described^13^. Based on receptor-ligand affinity, the reasonable binding models were selected according the score evaluated by London dG and GBVI/WAS dG score.

### Statistical analysis

All values were expressed as means ± SEM and analyzed using the statistical package for thesocial science (SPSS, version 15.0). Paired or unpaired two tailed t-tests were used to detect difference in the mean values of treatment group and control and analysis of variance (ANOVA) for the difference among more than two groups. Differences with P values < 0.05 were considered to be statistically significant

## Additional Information

**How to cite this article**: Zhang, Y. *et al.* Dietary component isorhamnetin is a PPARγ antagonist and ameliorates metabolic disorders induced by diet or leptin deficiency. *Sci. Rep.* **6**, 19288; doi: 10.1038/srep19288 (2016).

## Figures and Tables

**Table 1:** Sequences of the primers used in real-time PCR.

Gene	Forward primer	Reverse primer
β-Actin	TGTCCACCTTCCAGCAGATGT	AGCTCAGTAACAGTCCGCCTAGA
FAS	CTGAGATCCCAGCACTTCTTGA	GCCTCCGAAGCCAAATGAG
LPL	ATCGGAGAACTGCTCATGATGA	CGGATCCTCTCGATGACGAA
C/EBP-α	CGCAAGAGCCGAGATAAAGC	CACGGCTCAGCTGTTCCA
C/EBP-β	GGGGTTGTTGATGTTTTTGG	CGAAACGGAAAAGGTTCTCA
aP2	CATGGCCAAGCCCAACAT	CGCCCAGTTTGAAGGAAATC
ACC	GAATCTCCTGGTGACAATGCTTATT	GGTCTTGCTGAGTTGGGTTAGCT
CD36	GCTTGCAACTGTCAGCACAT	GCCTTGCTGTAGCCAAGAAC
PPARγ	TGCTGTATTTGAATCCGACGTT	GCTCTTTAGAAACTCCCTTGTCATG
SREBP-1c	GGCTATTCCGTGAACATCTCCTA	ATCCAAGGGCAGTTCTTGTG
SCD-1	TCACCTTGAGAGAAGAATTAGCA	TTCCCATTCCCTTCACTCTGA
UCP2	GGGCACTGCAAGCATGTGTA	TCAGATTCCTGGGCAAGTCACT
